# Survival analysis of elderly patients over 65 years old with stage II/III gastric cancer treated with adjuvant chemotherapy after laparoscopic D2 gastrectomy: a retrospective cohort study

**DOI:** 10.1186/s12885-021-07919-0

**Published:** 2021-02-25

**Authors:** Yanrui Liang, Liying Zhao, Hao Chen, Tian Lin, Tao Chen, Mingli Zhao, Yanfeng Hu, Jiang Yu, Hao Liu, Guoxin Li

**Affiliations:** Department of General Surgery, Nanfang Hospital, Southern Medical University, 1838 North Guangzhou Ave, Guangzhou, 510-515 China

**Keywords:** Gastric cancer, Gastrectomy, Adjuvant chemotherapy, Overall survival

## Abstract

**Background:**

The benefits of adjuvant chemotherapy for elderly patients with gastric cancer (GC) remain unknown because elderly patients are underrepresented in most clinical trials. This study aimed to evaluate the effectiveness and complications of adjuvant chemotherapy in patients > 65 years of age after laparoscopic D2 gastrectomy.

**Methods:**

This was a single-center retrospective cohort study of elderly patients (> 65 years) with stage II/III GC who underwent curative laparoscopic D2 gastrectomy with R0 resection between 2004 and 2018. The adjuvant chemotherapy regimens included monotherapy (oral capecitabine) and doublet chemotherapy (oral capecitabine plus intravenous oxaliplatin [XELOX] or intravenous oxaliplatin, leucovorin, and 5-fluorouracil [FOLFOX]). The data were retrieved from a prospectively registered database maintained at the Department of General Surgery in Nanfang Hospital, China. The patients were divided as surgery alone and surgery plus adjuvant chemotherapy (chemo group). The overall survival (OS), disease-free survival (DFS), chemotherapy duration, and toxicity were examined.

**Results:**

There were 270 patients included: 169 and 101 in the surgery and chemo groups, respectively. There were 10 (10/101) and six (6/101) patients with grade 3+ non-hematological and hematological adverse events. The 1−/3−/5-year OS rates of the surgery group were 72.9%/51.8%/48.3%, compared with 90.1%/66.4%/48.6% for the chemo group (log-rank test: *P* = 0.018). For stage III patients, the 1−/3−/5-year OS rates of the surgery group were 83.7%/40.7%/28.7%, compared with 89.9%/61.2%/43.6% for the chemo group (log-rank test: *P* = 0.015). Adjuvant chemotherapy was significantly associated with higher OS (HR = 0.568, 95%CI: 0.357–0.903, *P* = 0.017) and DFS (HR = 0.511, 95%CI: 0.322–0.811, *P* = 0.004) in stage III patients.

**Conclusions:**

This study suggested that adjuvant chemotherapy significantly improves OS and DFS compared with surgery alone in elderly patients with stage III GC after D2 laparoscopic gastrectomy, with a tolerable adverse event profile.

**Supplementary Information:**

The online version contains supplementary material available at 10.1186/s12885-021-07919-0.

## Background

Gastric cancer (GC), as the fifth most frequently diagnosed cancer and the third leading cause of cancer death worldwide, was responsible for over 1,000,000 new cases and an estimated 783,000 deaths in 2018 [1]. The markedly elevated incidence rates of GC in Eastern Asia (China, Japan, and Korea) indicate that GC is a significant public health threat, especially to the elderly, since over 60% of the GC diagnoses and 70% of GC-related mortality occur in elderly patients (aged 65 years or older) [2, 3]. As the population continues to age, the proportion of the population aged 60 years and over will increase from 12.4% in 2010 to 28% in 2040 [4]. Longer life expectancy also results in an increasing number of the elderly (aged 65 years or older) undergoing cancer operation and chemotherapy.

The survival benefits from gastrectomy plus chemotherapy have been confirmed in patients with advanced GC [5–7]. In the United States of America, chemoradiotherapy after gastrectomy has been confirmed to improve overall survival (OS) by the INT-0116 trial [8], while the progression-free survival (PFS) and OS benefits of perioperative chemotherapy have been shown by the MAGIC and FLOT4 trials [9, 10]. The adjuvant chemotherapy following D2 gastrectomy is a standard treatment for stage II/III GC in East Asia [11–13]. Although prior randomized controlled studies (RCTs) indicated that postoperative adjuvant treatment in patients who underwent D2 gastrectomy could improve the 5-year disease-free survival (DFS) and OS, the subgroup analyses showed that the survival benefits decreased with increasing age. Furthermore, the ACTS-GC study showed no statistically significant effects of postoperative chemotherapy on DFS and OS for patients older than 70 years (DFS: hazard ratios (HR) = 0.779, 95% confidence interval (CI): 0.527–1.151; OS: HR = 0.706, 95% CI: 0.490–1.017) [11]. Similar results for OS were observed in the CLASSIC study for patients older than 65 (HR = 0.70, 95% CI: 0.4–1.12) [12]. These results might be due to the considerably higher incidence of comorbidities, higher risk of complications, and shorter life expectancy of elderly patients [14]. Nevertheless, when considering those conflicting results, whether to give or not adjuvant chemotherapy to elderly patients with GC after D2 gastrectomy remains a dilemma for physicians. Therefore, the International Society of Geriatric Oncology suggested that specific trials for older patients with cancer should be conducted [15].

Laparoscopic gastrectomy has gained popularity worldwide for its safety and effectiveness [16, 17]. The Chinese Laparoscopic Gastrointestinal Surgery (CLASS) group recently reported the primary endpoints of the CLASS-01 trial, which suggested that laparoscopic distal gastrectomy for advanced gastric cancer was non-inferior to open surgery in terms of 3-year DFS and safety, with significant minimally invasive benefits [18, 19]. A previous study by our group indicated the potential benefits of laparoscopic gastrectomy for elderly patients with resectable GC [20]. Ushimaru et al. [21] reported that laparoscopic gastrectomy might improve the OS by reducing mortality from respiratory diseases. Still, the benefit of adjuvant chemotherapy after laparoscopic D2 gastrectomy in elderly patients is unknown.

As the elderly patients (over 65 years of age) are underrepresented in most RCTs, the present study aimed to evaluate the effectiveness and complications of adjuvant chemotherapy retrospectively in elderly patients (over 65 years of age) after laparoscopic D2 gastrectomy, based on a prospectively registered database in China. Those results could help shed some light on this controversy.

## Methods

### Study design and patients

This was a retrospective cohort study conducted in the Department of General Surgery of Nanfang Hospital in patients treated between June 2004 and June 2018. This study was approved by the Ethics Committee of Nanfang Hospital. The need for written informed consent was waived due to the retrospective nature of this study.

The inclusion criteria were: 1) over 65 years of age; 2) histologically confirmed stage II or III gastric adenocarcinoma, according to American Joint Committee on Cancer (AJCC, 7th Edition); 3) received curative laparoscopic gastrectomy with D2 nodal dissection at Nanfang Hospital; and 4) at least 15 lymph nodes were available to ensure adequate disease classification. The exclusion criteria were: 1) residual tumors (R1/R2 resections or palliative surgery; 2) death within 1 month after surgery; 3) a previous history of primary or secondary tumor beside the current GC; 4) neoadjuvant chemotherapy or adjuvant radiotherapy; or 5) incomplete medical record .

The enrolled patients were divided into surgery alone group (surgery group) and the surgery plus adjuvant chemotherapy group (chemo group).

### Adjuvant chemotherapy protocols

The adjuvant chemotherapy regimens administered during the study period included monotherapy and doublet chemotherapy. The initial dose of each regimen was reduced to 75% of the original value to minimize toxic effects in elderly patients. For monotherapy, the patients received 3-week cycles of oral capecitabine (750 mg/m^2^ twice daily on days 1 to 14 of each cycle) for 6 months if tolerated. For doublet chemotherapy, the patients received oral capecitabine plus intravenous oxaliplatin (XELOX) or 2-week cycles of intravenous oxaliplatin, leucovorin, and 5-fluorouracil (FOLFOX). For the XELOX regimen, patients received 3-week cycles of oral capecitabine (750 mg/m^2^ twice daily on days 1 to 14 of each cycle) plus intravenous oxaliplatin (130 mg/m^2^ on day 1 of each cycle) for 6 months if tolerated. The FOLFOX regimen was administered as follows: intravenous (IV) treatment with 63.75 mg/m^2^ of oxaliplatin, 300 mg/m^2^ of leucovorin and IV push administration of 300 mg/m^2^ of fluorouracil on day 1, and 900 mg/m^2^ of fluorouracil IV by continuous infusion for 24 h on days 1 and 2. This regimen was repeated every 14 days and lasted for 6 months if tolerated. In all regimens, the dose of each drug was reduced to the next lower dose increment in case of grade 4 neutropenia or thrombocytopenia, or grade 3 or above febrile neutropenia.

### Data collection

The data were retrieved from a prospectively registered database maintained at the Department of General Surgery in Nanfang Hospital, China. The database includes patient characteristics (age at diagnosis, sex, etc.), clinical variables (postoperative stay, circulating tumor cells [CTCs] collected at postoperative two weeks, etc.), pathological features (tumor size, histological grade, etc.), chemotherapy (regimen, duration, cycles of chemotherapy, and grade 3 or above toxicity events), and follow-up. All data are routinely updated after each routine follow-up visit, either at the outpatient clinic or by phone. For the present study, the last follow-up data were collected on May 30th, 2018.

### Outcomes

The observation outcomes of this study were OS and DFS. The OS was calculated from the date of operation to either the date of death or the date of the last follow-up visit. The DFS was calculated from the date of resection to the date of the first recurrence detected, or the last follow-up visit. Recurrence was determined as the appearance of any new lesion either locally, regionally, or distant. All grade 3 or above hematological and non-hematological toxicity events were recorded. Toxicities were graded according to the National Cancer Institute common toxicity criteria version 3.0 [22].

### Statistical analysis

All statistical analyses were performed using SPSS 24.0 (IBM Corp., Armonk, NY, USA). Continuous variables were tested for normal distribution using the Kolmogorov-Smirnov test. Those with a normal distribution were expressed as means ± standard deviations (SD) and were analyzed using Student’s t-test; otherwise, they were presented as medians (ranges) and analyzed using the Mann-Whitney U-test. Categorical variables were reported as numbers with percentages and analyzed using the chi-square test with the Yates correction or Fisher’s exact test, as appropriate. The OS and DFS rates were compared between the surgery and chemo groups using unadjusted Kaplan-Meier curves and the log-rank test. The OS and DFS rates of different clinicopathological characteristics and different chemotherapy regimen were compared. HR and 95% CI were used to estimate the role of each independent predictor of survival. The Cox regression model was used for univariable and multivariable analyses. We adjusted for the following variables: treatment regimens (with adjuvant chemotherapy or not), age of diagnosis, time of surgery, sex, ECOG score, Charlson score, tumor location, retrieved lymph node, hospital stays, AJCC stage, histologic grade, lymphatic, blood vessel or perineural invasion, tumor size, and CTC counts. Variables with *P* < 0.1 in the univariable analysis were included in the multivariable analysis. The level of significance was set at a two-tailed *P*-value of 0.05.

## Results

### Patient characteristics

**Fig. 1:**
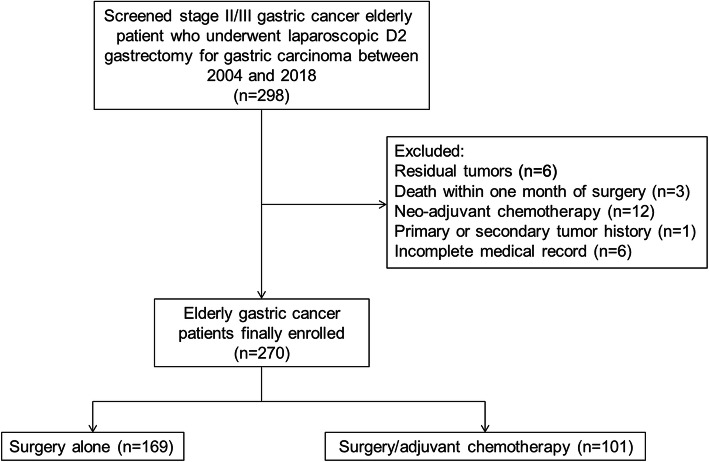
Patient flowchart

At first, 298 patients were eligible, but 28 were excluded because of the presence of residual tumors (R1/R2 resections and palliative surgery), death within 1 month of surgery, primary or secondary tumor history, neoadjuvant chemotherapy, adjuvant radiotherapy, or incomplete medical record. The remaining 270 patients were included in the analysis; 169 and 101 were classified in the surgery and chemo groups, respectively (Fig. 1).

Table 1 presents the characteristics of the patients. The median age in the surgery and chemo groups was 70 years (69–75 years) and 69 years (66–72 years), respectively (*P* = 0.001). The male-to-female ratio was 2.1:1 in the surgery group and 3.2:1 in the chemo group (*P* = 0.152). Most patients in the two groups had an ECOG score and a Charlson comorbidity score of < 1 (ECOG: 88.2 and 89.1%; Charlson score: 94.7 and 96.0%; all *P* > 0.05). The number of CTCs in the surgery group was 5 (2–16), while 4 (1–7) in the chemo group (*P* = 0.328). In the surgery group, there were 56 patients with AJCC stage II and 113 with AJCC stage III, while in the chemo group, there were 25 patients with AJCC stage II and 76 with AJCC stage III (*P* = 0.147). In the chemo group, six patients received monotherapy, and 95 patients received platinum-based doublet chemotherapy therapy; 57 patients received adjuvant chemotherapy for less than 3 months, while 44 patients received 3–6 months of adjuvant chemotherapy. The median follow-up in the surgery and chemo groups was 25 (IQR 13–44) months, and 22 (IQR 11–54.5) months, respectively (*P* = 0.452).

**Table 1:** Baseline information of patients

Characteristics	Surgery *N* = 169	Chemotherapy *N* = 101	*p*
**Clinical characteristics**			
Age at diagnosis (years), median (IQR)	70 (67–75)	69 (66–72)	0.001
65–70, *n* (%)	92 (54.4)	64 (63.4)	0.152
> 70, *n* (%)	77 (45.6)	40 (39.6)	
Time of surgery, *n* (%)			0.684
2005–2014	81 (47.9)	51 (50.5)	
2015–2018	88 (52.1)	50 (49.5)	
Sex, *n* (%)			0.152
Male	115 (68.0)	77 (76.2)	
Female	54 (32.0)	24 (23.8)	
ECOG score			0.281
0	78 (46.2)	54 (63.5)	
1	71 (42.0)	36 (35.6)	
2	14 (8.3)	9 (8.9)	
2+	6 (3.5)	2 (2.0)	
Charlson score			0.711
0	128 (75.7)	74 (73.3)	
1	32 (18.9)	23 (22.8)	
2	8 (4.8)	3 (2.9)	
2+	1 (0.6)	1 (1.0)	
Tumour location			0.524
Gastroesophageal junction	58 (34.3)	39 (38.6)	
Antrum	88 (52.1)	49 (48.5)	
Other	23 (13.6)	13 (12.9)	
Hospital stays (days), median (IQR)	11 (8–16)	10.0 (8–15)	0.328
CTC (number), median (IQR)	5 (2–16)	4 (1–7)	0.270
**Pathological characteristics**			
TMN stage, *n* (%)			0.147
II	56 (33.1)	25 (24.8)	
III	113 (66.9)	76 (75.2)	
T stage, *n* (%)			0.286
T1–2	13 (7.7)	5 (5.0)	
T3	35 (20.7)	18 (17.8)	
T4	121 (71.6)	78 (77.2)	
N stage, *n* (%)			0.285
N0	45 (26.6)	17 (16.8)	
N1	18 (10.7)	17 (16.8)	
N2	44 (26.0)	26 (25.8)	
N3	62 (36.7)	41 (40.6)	
Retrieved lymph nodes (number), median (IQR)	40 (25–55.5)	38 (24–54)	0.516
Grade, *n* (%)			0.051
Well or moderately differentiated	123 (72.8)	84 (83.2)	
Poorly differentiated or undifferentiated	46 (27.2)	17 (16.8)	
Lymphatic, blood vessel or perineural invasion, *n* (%)	100 (59.2)	62 (61.4)	0.720
Tumour size (cm), mean ± SD	4.0 (3.0–5.5)	4.5 (3.2–6.0)	0.121
≤ 5 cm	104 (61.5)	51 (50.5)	0.076
> 5 cm	65 (38.5)	50 (49.5)	
**Drug delivery and toxicities**			
Adjuvant chemotherapy regimen			
Mono chemotherapy		6 (5.9)	
Doublet chemotherapy		95 (94.1)	
Length of adjuvant chemotherapy			
< 3 months		57 (56.4)	
3–6 months		44 (43.6)	
Toxicities (grade 3 or more)			
Monotherapy		1 (1.0)	
Non-hematological adverse events		1 (1.0)	
Hematological adverse events		0	
Double therapy		15 (14.9)	
Non-hematological adverse events		9 (8.9)	
Hematological adverse events		6 (5.9)	

*IQR* interquartile range, *ECOG* Eastern Cooperative Oncology Group, *CTC* circulating tumor cells

### Factors associated with OS and DFS in all patients

The univariable and multivariable Cox proportional hazards models for all patients are shown in Table 2. In the multivariable analysis, age > 70 years (HR = 1.640, 95% CI: 1.119–2.403, *P* = 0.011) and stage III GC (HR = 2.738, 95% CI: 1.677–4.471, *P* < 0.001) were independently associated with OS. Surgery plus adjuvant chemotherapy (HR = 0.511, 95% CI: 0.322–0.811, *P* = 0.004), surgery performed in 2015–2018 (HR = 0.586, 95% CI: 0.376–0.912, *P* = 0.018), and stage III GC (HR = 2.345, 95% CI: 1.466–3.751, *P* < 0.001) were independently associated with DFS. Therefore, stage III GC was independently associated with both OS and DFS.

**Table 2:** Association factors of OS and DFS in the total patients

	Overall survival				Disease-free survival			
	Univariable analysis		Multivariable analysis		Univariable analysis		Multivariable analysis	
Factors	HR (95% CI)	*p*	HR (95% CI)	*p*	HR (95% CI)	*p*	HR (95% CI)	*p*
Treatment								
Surgery alone	1 (Reference)	–			1 (Reference)	–	1 (Reference)	–
Surgery/adjuvant chemotherapy	0.733 (0.486–1.106)	0.139			0.673 (0.447–1.012)	0.057	0.511 (0.322–0.811)	0.004
Age of diagnosis								
65–70 years	1 (Reference)	–	1 (Reference)	–	1 (Reference)	–		
> 70 years	1.560 (1.021–2.385)	0.028	1.640 (1.119–2.403)	0.011	1.422 (0.978–2.067)	0.065		
Time of surgery								
2004–2014	1 (Reference)	–			1 (Reference)	–	1 (Reference)	–
2015–2018	0.967 (0.632–1.478)	0.876			0.713 (0.475–1.069)	0.102	0.586 (0.376–0.912)	0.018
Sex								
Male	1 (Reference)	–			1 (Reference)	–		
Female	0.903 (0.589–1.383)	0.638			0.895 (0.588–1.361)	0.603		
ECOG score								
0	1 (Reference)	–			1 (Reference)	–		
1	1.325 (0.879–1.996)	0.179			1.468 (0.982–2.194)	0.062		
2	1.006 (0.504–2.008)	0.987			1.105 (0.554–2.202)	0.777		
2+	2.016 (0.797–5.100)	0.139			2.017 (0.798–5.097)	0.138		
Charlson score								
0	1 (Reference)	–			1 (Reference)	–		
1	1.029 (0.631–1.679)	0.909			1.068 (0.661–1.724)	0.788		
2	0.992 (0.363–2.710)	0.988			1.182 (0.479–2.961)	0.716		
2+	1.135 (0.158–8.174)	0.900			1.368 (0.190–9.852)	0.756		
Tumor location								
Gastroesophageal junction	1 (Reference)	–			1 (Reference)	–		
Antrum	1.304 (0.841–2.023)	0.236			1.321 (0.861–2.027)	0.203		
Other	1.511 (0.832–2.724)	0.175			1.497 (0.840–2.670)	0.171		
Retrieved lymph node	1.002 (0.9931.011)	0.656			0.999 (0.990–1.008)	0.841		
Hospital stays	1.003 (0.983–1.023)	0.793			1.006 (0.987–1.025)	0.550		
AJCC stage								
II	1 (Reference)	–	1 (Reference)	–	1 (Reference)	–	1 (Reference)	–
III	2.626 (1.610–4.284)	< 0.001	2.738 (1.677–4.471)	< 0.001	2.345 (1.466–3.751)	< 0.001	2.345 (1.466–3.751)	< 0.001
Histologic Grade								
Well or moderately differentiated	1 (Reference)	–			1 (Reference)	–		
Poorly differentiated or undifferentiated	1.293 (0.809–2.068)	0.283			1.247 (0.788–1.975)	0.346		
Lymphatic, blood vessel or perineural invasion								
Yes	1 (Reference)	–			1 (Reference)	–		
No	0.857 (0.581–1.7266	0.439			0.740 (0.505–1.083)	0.121		
Tumor size								
≤ 5 cm	1 (Reference)	–	1 (Reference)		1 (Reference)	–		
> 5 cm	1.610 (1.099–2.356)	0.014	1.356 (0.921–1.996)	0.123	1.440 (0.991–2.091)	0.560		
CTC	0.937 (0.802–1.096)	0.416			1.007 (0.924–1.088)	0.866		

*HR* hazards ratio, *CI* confidence interval, *ECOG* Eastern Cooperative Oncology Group, *AJCC* American Joint Cancer Committee, *CTC* circulating tumor cells

We adjusted for the following variables: treatment regimens (with adjuvant chemotherapy or not), age of diagnosis, time of surgery, sex, ECOG score, Charlson score, tumor location, retrieved lymph node, hospital stays, AJCC stage, histologic grade, lymphatic, blood vessel or perineural invasion, tumor size, and CTC counts

### Overall survival

**Fig. 2:**
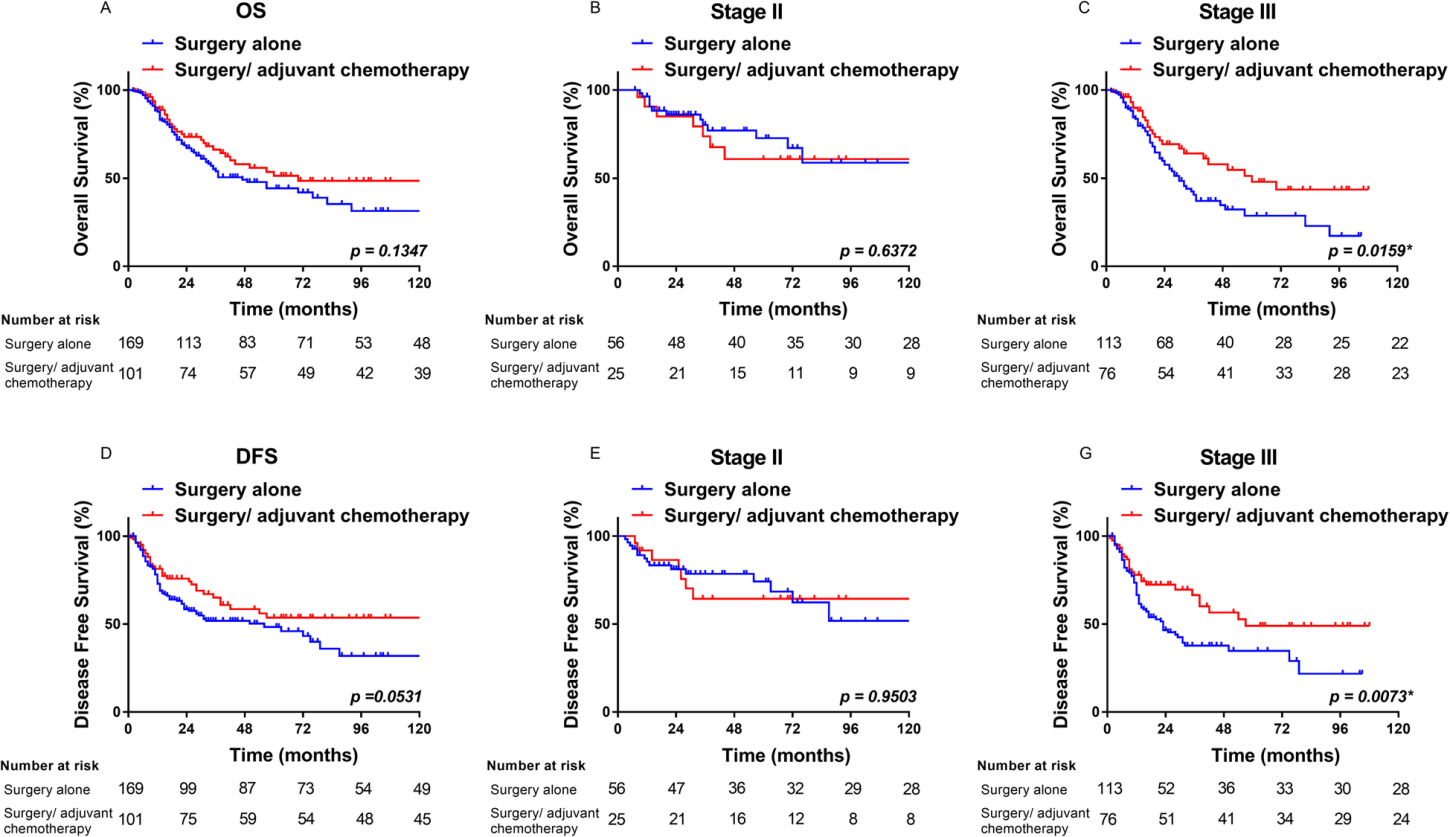
Survival curves for overall survival (OS) and disease-free survival (DFS) in all the patients **a**, **d**, stage II patients **b**, **e**, and stage III patients **c**, **f**

Unadjusted Kaplan-Meier survival curves were constructed for all patients in the two groups. The 1-, 3-, and 5-year OS rates of the surgery group were 72.9, 51.8, and 48.3%, compared with 90.1, 66.4, and 48.6% for the chemo group, respectively (HR = 0.61, 95% CI: 0.42–0.92, *P* = 0.135) (Fig. 2a). In the stage II cohort, the 1-, 3-, and 5-year OS rates of the surgery group were 96.3, 80.4, and 72.7%, compared with 90.5, 73.8, and 50.9% for the chemo group, respectively (HR = 1.26, 95% CI: 0.483–3.29, *P* = 0.637) (Fig. 2b). For stage III patients, the 1-, 3-, and 5-year OS rates of the surgery group were 83.7, 40.7, and 28.7%, compared with 89.9, 61.2, and 43.6% for the chemo group, respectively (HR = 0.58, 95% CI: 0.38–0.90, *P* = 0.016) (Fig. 2c).

### Disease-free survival

The 1-, 3-, and 5-year DFS rates of the surgery group were 72.9, 51.8, and 48.4%, compared with 81.3, 65.1, and 53.6% for the chemo group, respectively (HR = 0.682, 95% CI: 0.463–1.005, *P* = 0.053) (Fig. 2d). In the stage II cohort, the 1-, 3-, and 5-year DFS rates of the surgery group were 85.3, 78.5, and 74.2%, compared with 91.8, 64.4, and 64.4% for the chemo group, respectively (HR = 1.02, 95% CI: 0.416–2.54, *P* = 0.950 (Fig. 2e). In stage III patients, the 1-, 3-, and 5-year DFS rates of the surgery group were 66.5, 37.7, and 34.8%, compared with 77.9, 60.0, and 49.0% for the chemo group, respectively (HR = 0.55, 95% CI: 0.36–0.83, *P* = 0.007) (Fig. 2f). The OS and DFS in the stage III subgroup were significantly different between the surgery and Chemo groups.

### Subgroup survival analysis in stage III patients

The univariable and multivariable Cox proportional hazards models in stage III patients are shown in Table 3. Surgery plus adjuvant chemotherapy (HR = 0.568, 95% CI: 0.357–0.903, *P* = 0.017) and age > 70 years (HR = 1.573, 95% CI: 1.029–2.405, *P* = 0.036) were independently associated with OS. Surgery plus adjuvant chemotherapy (HR = 0.511, 95% CI: 0.322–0.811, *P* = 0.004) and surgery performed in 2015–2018, HR = 0.586, 95% CI: 0.376–0.912, *P* = 0.018) were independently associated with DFS.

**Table 3:** Association factors of OS and DFS in stage III patients

	Overall survival				Disease-free survival			
	Univariable analysis		Multivariable analysis		Univariable analysis		Multivariable analysis	
Factors	HR (95% CI)	*p*	HR (95% CI)	*p*	HR (95% CI)	*p*	HR (95% CI)	*p*
Treatment								
Surgery alone	1 (Reference)	–	1 (Reference)	–	1 (Reference)	–	1 (Reference)	–
Surgery/adjuvant chemotherapy	0.572 (0.360–0.910)	0.018	0.568 (0.357–0.903)	0.017	0.542 (0.342–0.859)	0.009	0.511 (0.322–0.811)	0.004
Age of diagnosis								
65–70 years	1 (Reference)	–	1 (Reference)	–	1 (Reference)	–		
> 70 years	1.560 (1.021–2.385)	0.040	1.573 (1.029–2.405)	0.036	1.409 (0.926–1.943)	0.109		
Time of surgery								
2004–2014	1 (Reference)	–			1 (Reference)	–	1 (Reference)	–
2015–2018	0.895 (0.565–1.417)	0.636			0.630 (0.405–0.980)	0.041	0.586 (0.376–0.912)	0.018
Sex								
Male	1 (Reference)	–			1 (Reference)	–		
Female	0.987 (0.609–1.602)	0.959			0.957 (0.591–1.55)	0.860		
ECOG score								
0	1 (Reference)	–			1 (Reference)	–		
1	1.345 (0.848–2.132)	0.207	–	–	1.493 (0.948–2.351)	0.083		
2	1.201 (0.576–2.507)	0.625			1.335 (0.643–2.773)	0.438		
2+	2.810 (1.098–7.188)	0.031			2.715 (1.061–6.950)	0.037		
Charlson score								
0	1 (Reference)	–			1 (Reference)	–		
1	1.038 (0.609–1.771)	0.890			1.119 (0.664–1.885)	0.674		
2	1.000 (0.364–2.749)	0.999			1.186 (0.477–2.946)	0.713		
2+	–	–						
Tumor location								
Gastroesophageal junction	1 (Reference)	–			1 (Reference)	–		
Antrum	1.347 (0.838–2.166)	0.219			1.368 (0.858–2.183)	0.188		
Other	1.862 (0.917–3.780)	0.086			1.521 (0.753–3.073)	0.242		
Retrieved lymph node	1.001 (0.991–1.010)	0.853			0.999 (0.989–1.009)	0.782		
Hospital stays	1.001 (0.980–1.023)	0.905			1.005 (0.986–1.025)	0.600		
T stage								
T1–2	1 (Reference)	–			1 (Reference)	–		
T3	0.596 (0.130–2.729)	0.505			0.464 (0.103–2.098)	0.318		
T4	0.722 (0.176–2.958)	0.651			0.576 (0.141–2.358)	0.443		
N stage								
N0	1 (Reference)	–			1 (Reference)	–		
N1	0.223 (0.040–1.231)	0.085			0.234 (0.042–1.288)	0.095		
N2	0.517 (0.121–2.207)	0.373			0.555 (0.131–2.357)	0.425		
N3	1.071 (0.261–4.398)	0.924			1.065 (0.259–4.375)	0.930		
Histologic Grade								
Well or moderately differentiated	1 (Reference)	–			1 (Reference)	–		
Poorly differentiated or undifferentiated	1.035 (0.609–1.759)	0.899			1.043 (0.615–1.769)	0.877		
Lymphatic, blood vessel or perineural invasion								
Yes	1 (Reference)	–			1 (Reference)	–		
No	0.899 (0.581–1.389)	0.631			0.741 (0.484–1.134)	0.167		
Tumor size								
≤ 5 cm	1 (Reference)	–			1 (Reference)	–		
> 5 cm	1.209 (0.793–1.843)	0.377			1.147 (0.756–1.738)	0.519		

*HR* hazards ratio, *CI* confidence interval, *ECOG* Eastern Cooperative Oncology Group, *AJCC* American Joint Cancer Committee

We adjusted for the following variables: treatment regimens (with adjuvant chemotherapy or not), age of diagnosis, time of surgery, sex, ECOG score, Charlson score, tumor location, retrieved lymph node, hospital stays, T stage, N stage, histologic grade, lymphatic, blood vessel or perineural invasion, and tumor size

### Chemotherapy regimens, duration, and toxicity

 In stage III patients, the platinum-based doublet chemotherapy led to better OS and DFS compared with monotherapy (OS: *P* = 0.037; DFS: *P* = 0.013) (Supplementary Fig. S2 A, C), but the differences were not statistically significant in stage II patients (*P* = 0.473 and *P* = 0.499) (Supplementary Fig. S1A, C). No significant differences in OS and DFS were observed in relation to chemotherapy duration (all *P* > 0.05) (Supplementary Fig. S1 B,D; Supplementary Fig. S2 B, D). There were 10 patients with grade 3 or above non-hematological toxicity adverse events, and six with grade 3 hematological toxicity adverse events (neutropenia) (Table 1).

### Analysis in patients with available CTC data

Forty-three patients had a CTC count before surgery, and 40 of them were positive. There were no significant differences in OS and DFS between the surgery and chemo groups among CTC-tested patients (Supplementary Fig. S3 A, C) and CTC-positive patients (all *P* > 0.05) (Supplementary Fig. S3 B,D).

## Discussion

The benefits of adjuvant chemotherapy for elderly patients (age over 65) with GC remain unknown because the elderly patients are underrepresented in most clinical trials [15]. Therefore this study aimed to evaluate the effectiveness and complications of adjuvant chemotherapy in elderly patients (over 65 years of age) after laparoscopic D2 gastrectomy. The results strongly suggest that adjuvant chemotherapy improves the OS and DFS of elderly patients with stage III GC operated using D2 laparoscopic gastrectomy compared with surgery alone.

Previously, there were a few single-center retrospective studies that focused on adjuvant chemotherapy for elderly patients after gastrectomy [23, 24]. Still, those previous studies might not represent the current status of advanced GC treatment since laparoscopic D2 gastrectomy became popular relatively recently [18]. In the present study, only elderly gastric patients who underwent laparoscopic D2 gastrectomy were included. Among them, 41% received adjuvant chemotherapy in the 65–70 age group and only 33% in the > 70 age group. This finding is similar to other cancers [25, 26]. This may be due to two reasons. First, there is no solid evidence to prove the efficacy of adjuvant chemotherapy in elderly patients with GC. Second, with a high comorbidity rate, older patients may prefer not to undergo chemotherapy treatment in their relatively limited lifetime [27].

Chemotherapy toxicity is another concern of the elderly who just underwent surgery. In the CLASSIC study, 56% of the patients who received the fluoropyrimidine-platinum chemotherapy regimen experienced grade 3–4 adverse events [12]. In the ACTS-GC study, 22.8% of patients with mono-chemotherapy experienced grade 3–4 adverse events [11]. In the present study, 95 (94.0%) patients in the chemo group received platinum-based doublet chemotherapy, including XELOX and FOLFOX, and 15 (15.6%) patients suffered from grade 3–4 adverse events. In the monotherapy group, one patient suffered from grade 3–4 non-hematological adverse events. The adverse event rate in our cohort is similar to a retrospective study from Korea [24]. Low rates of grade 3–4 adverse events may be due to the low Charlson comorbidity score in the present study since the patients were required to be able to tolerate laparoscopic D2 gastrectomy. The result indicates that adjuvant chemotherapy is tolerable in elderly patients who were suitable for gastrectomy. Still, it is possible that the adverse events were underestimated or not measured strictly in this retrospective study.

In the present study, adjuvant chemotherapy could significantly improve the OS in stage III elderly patients. Jin et al. [23] revealed an OS benefit (*P* = 0.003) of adjuvant chemotherapy in elderly patients in a single-center retrospective study. A single-center retrospective study of elderly patients with GC (over 70 years) in Korea reported a DFS benefit (*P* = 0.03) after adjuvant chemotherapy, but without an OS benefit (*P* = 0.242, 24]. Nevertheless, by analyzing elderly patients with resected GC in the SEER-Medicare database, Hoffman et al. [28] reported that elderly patients might not gain a survival benefit from adjuvant chemotherapy, but most cases in this database underwent D0 or D1 gastrectomy. Up to now, no standard adjuvant chemotherapy regimens were established for the elderly. Some reports suggest that patients might benefit from adjuvant chemotherapy, no matter which chemotherapy regimen is used [29]. The CLASSIC study indicated that the fluoropyrimidine and oxaliplatin combination reduced both locoregional and distant recurrences, but had a smaller effect on peritoneal recurrences [12]. Kim et al. [30] reported that there were no significant improvements in OS and RFS when using longer treatments of fluoropyrimidine-based adjuvant chemotherapy in patients with stage II or III GC. Similar results were also observed in stage III colon cancer with 3 vs. 6 months of XELOX [31]. On the other hand, Feng et al. [32] reported that additional oral capecitabine for 6 months after eight cycles of XELOX improved the DFS and OS for stage IIIA GC. Still, those previous studies were not focused on elderly patients with GC. Elderly patients may prefer to undergo fewer treatments or treatments with fewer adverse effects in their relatively limited lifetime [27].

There are several limitations to this study. First, this study was based on retrospective data, with inherent shortcomings. For example, immortal time bias in the adjuvant group could not be completely avoided in a retrospective study. Secondly, it was a single center study, and it is unknown whether the results are valid externally. In addition, this was a strictly selected group of patients, excluding those with previous cancers, R1/2 resections and post-operative death. Consequently, the survival rates in both groups might not reflect real-world data. Finally, differences between the < 65 and ≥ 65 year-old groups were not assessed. Further prospective studies are needed to address those issues.

## Conclusions

In this retrospective, single-institution study, the OS and DFS benefited from adjuvant chemotherapy in elderly patients with stage III GC after D2 laparoscopic gastrectomy. Well-designed prospective studies are needed to confirm these findings. Elderly patients are highly variable in their functional status and comorbidities. Thus, cofactors regarding the functional, social, and mental status should also be considered. Further studies are needed to identify the elderly who can tolerate and benefit from adjuvant chemotherapy.

## Contribution to the field statement

The benefits of adjuvant chemotherapy for elderly patients (age > 65) with gastric cancer (GC) remain unknown because elderly patients are underrepresented in most clinical trials. A total of 270 patients included for analysis. There were ten (10/101) and six (6/101) patients with grade 3+ non-hematological and hematological adverse events. The 1−/3−/5-year OS rates of the surgery group were 72.9%/51.8%/48.3%, compared with 90.1%/66.4%/48.6% for the chemo group (log-rank test: *P* = 0.018). For stage III patients, the 1−/3−/5-year OS rates of the surgery group were 83.7%/40.7%/28.7%, compared with 89.9%/61.2%/43.6% for the chemo group (log-rank test: *P* = 0.015). Adjuvant chemotherapy was significantly associated with higher OS (HR = 0.568, 95%CI: 0.357–0.903, *P* = 0.017) and DFS (HR = 0.511, 95%CI: 0.322–0.811, *P* = 0.004) in stage III patients. CTC > 0 had no significant impact on the benefits of adjuvant chemotherapy on OS and DFS. These findings suggested that adjuvant chemotherapy significantly improves OS and DFS for elderly patients with stage III GC after D2 laparoscopic gastrectomy, with a tolerable adverse event profile.

## Supplementary Information

**Additional file 1: Supplementary Figure S1.** Subgroup survival analysis for stage II patients in the chemotherapy group.

**Additional file 2; Supplementary Figure S2.** Subgroup survival analysis for stage III patients in the chemotherapy group.

**Additional file 3: Supplementary Figure S3.** Subgroup survival analysis in patients who had been tested for circulating tumor cells (CTCs) (A, C), and for those with positive CTCs (B, D).

## Data Availability

The datasets used and analysed during the current study are available from the corresponding author on reasonable request.

## Abbreviations

GC: Gastric cancer
OS: Overall survival
DFS: Disease-free survival
CTCs: Circulating tumor cells
PFS: Progression-free survival
RCTs: Randomized controlled studies
HR: Hazard ratios
CI: Confidence interval
CLASS: The chinese laparoscopic gastrointestinal surgery
AJCC: American Joint Committee on Cancer
SD: Standard deviations
