# Correction to: Survival analysis of elderly patients over 65 years old with stage II/III gastric cancer treated with adjuvant chemotherapy after laparoscopic D2 gastrectomy: a retrospective cohort study

**DOI:** 10.1186/s12885-021-08037-7

**Published:** 2021-03-23

**Authors:** Yanrui Liang, Liying Zhao, Hao Chen, Tian Lin, Tao Chen, Mingli Zhao, Yanfeng Hu, Jiang Yu, Hao Liu, Guoxin Li

**Affiliations:** Department of General Surgery, Nanfang Hospital, Southern Medical University, 1838 North Guangzhou Ave, Guangzhou, 510-515 China

**Correction to: BMC Cancer 21, 196 (2021)**

**https://doi.org/10.1186/s12885-021-07919-0**

Following publication of the original article [1], the authors reported that the incorrect corresponding author had been assigned. The correct corresponding authors are Guoxin Li and Hao Liu.
