# Supplementary figures and images for: Survival analysis of elderly patients over 65 years old with stage II/III gastric cancer treated with adjuvant chemotherapy after laparoscopic D2 gastrectomy: a retrospective cohort study
Source: BMC Cancer. 2021 Feb 25;21:196. doi: 10.1186/s12885-021-07919-0 (PMC7908711; doi:10.1186/s12885-021-07919-0)

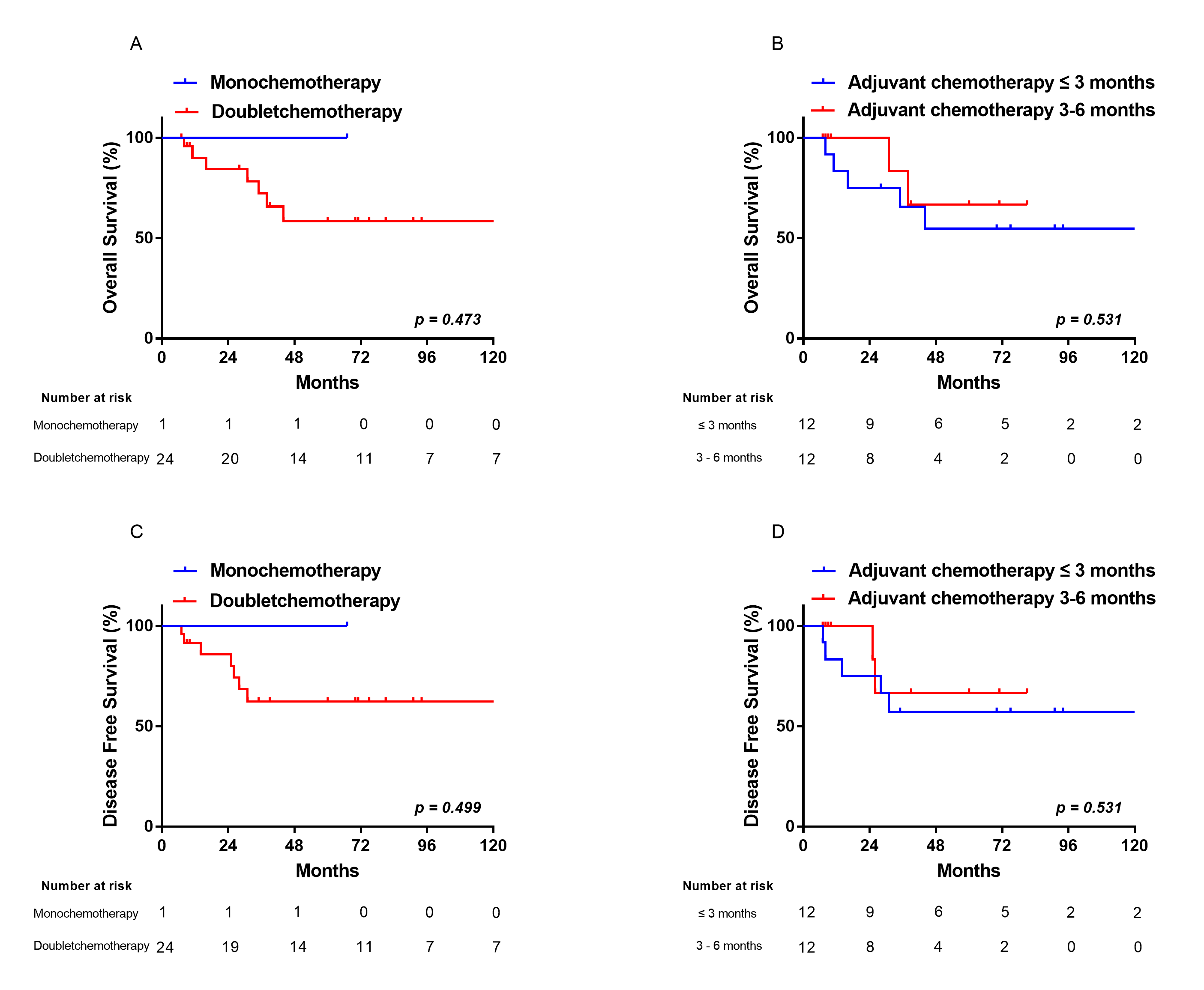

Supplement: Supplementary file 1 — Additional file 1: Supplementary Figure S1. Subgroup survival analysis for stage II patients in the chemotherapy group. [file 12885_2021_7919_MOESM1_ESM.tif]

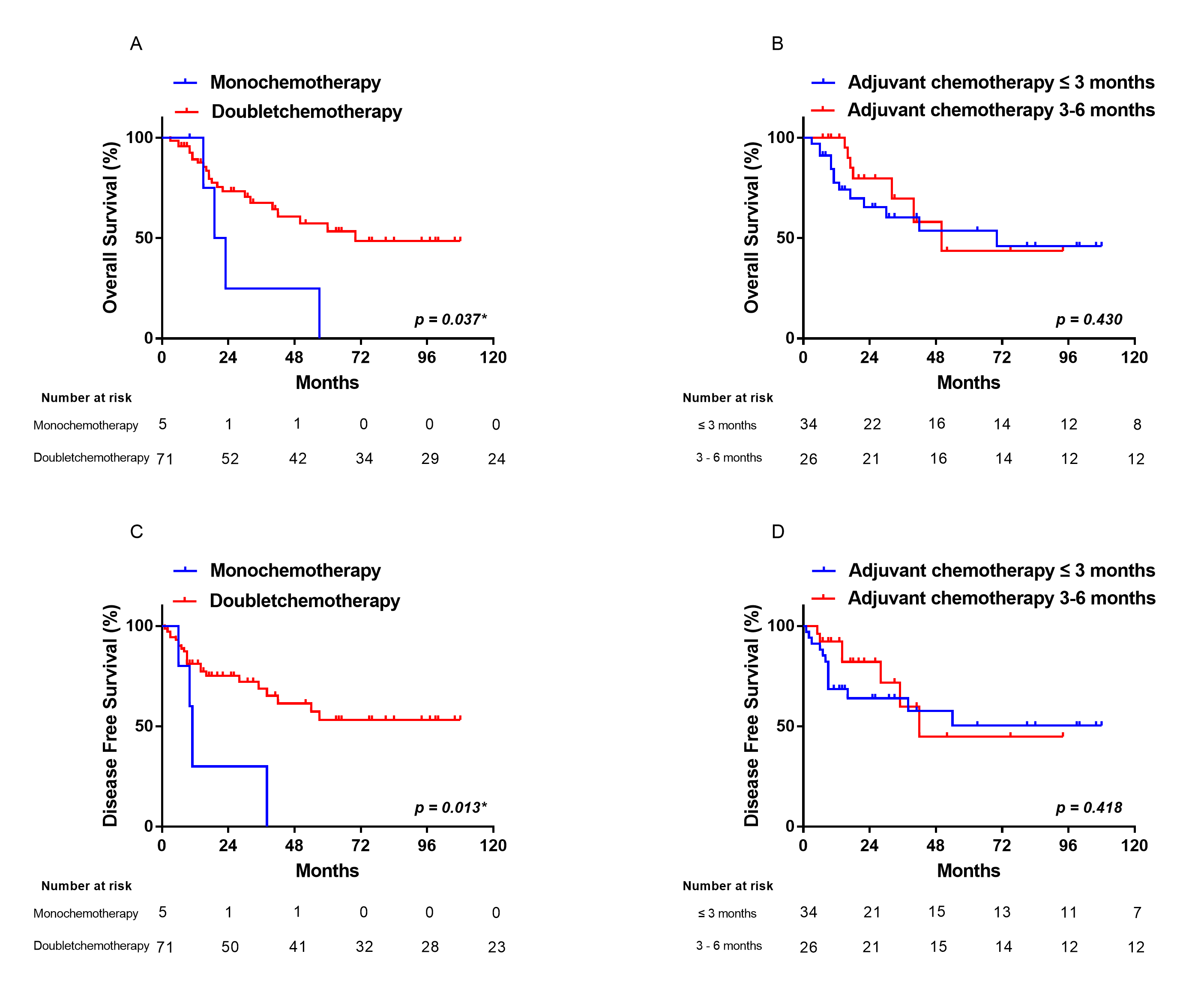

Supplement: Supplementary file 2 — Additional file 2; Supplementary Figure S2. Subgroup survival analysis for stage III patients in the chemotherapy group. [file 12885_2021_7919_MOESM2_ESM.tif]

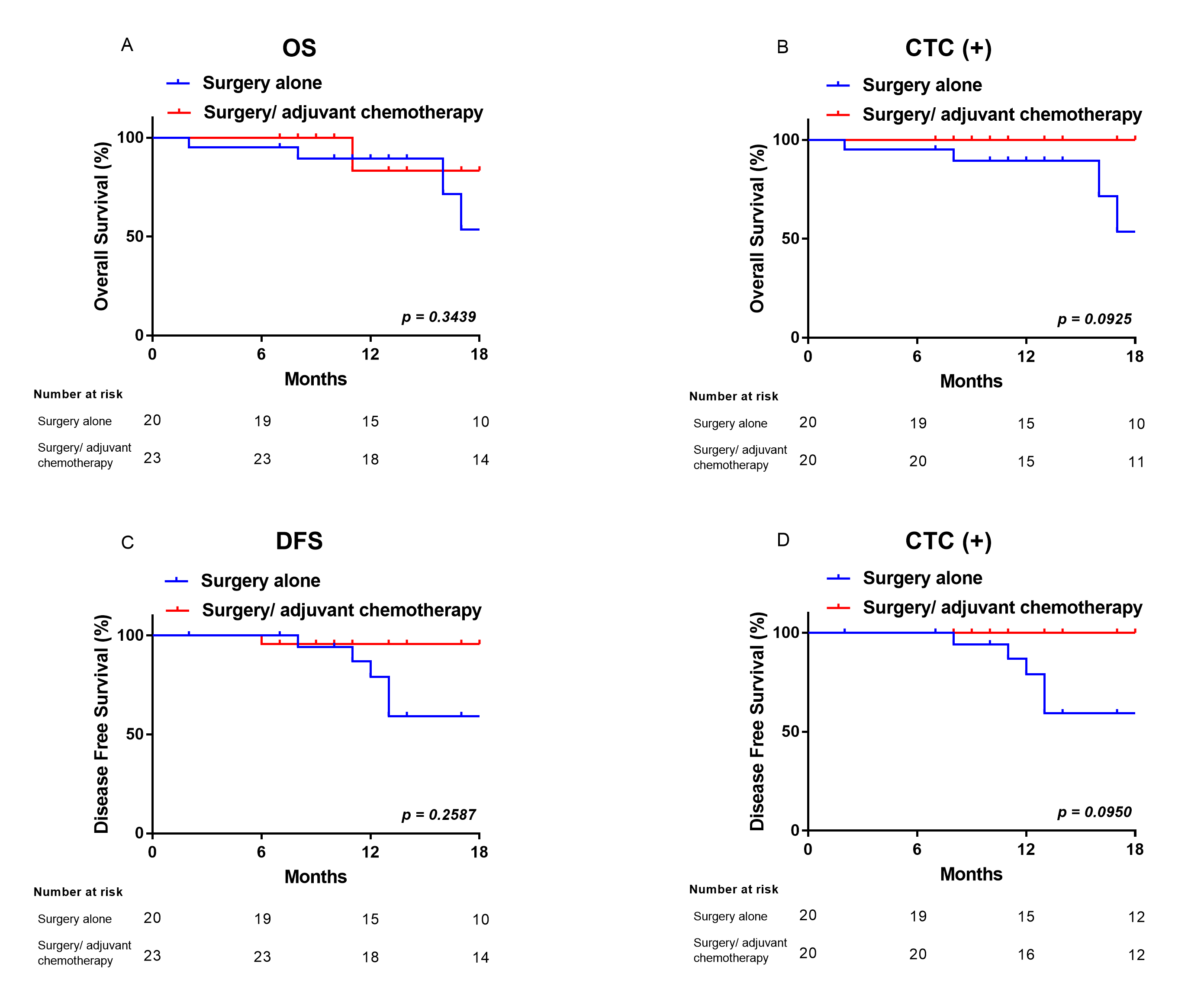

Supplement: Supplementary file 3 — Additional file 3: Supplementary Figure S3. Subgroup survival analysis in patients who had been tested for circulating tumor cells (CTCs) (A, C), and for those with positive CTCs (B, D). [file 12885_2021_7919_MOESM3_ESM.tif]
